# How can interventions that target forest-goers be tailored to accelerate malaria elimination in the Greater Mekong Subregion? A systematic review of the qualitative literature

**DOI:** 10.1186/s12936-019-2666-5

**Published:** 2019-02-01

**Authors:** Stephanie D. Nofal, Thomas J. Peto, Bipin Adhikari, Rupam Tripura, James Callery, Thanh Mai Bui, Lorenz von Seidlein, Christopher Pell

**Affiliations:** 1 Faculty of Infectious and Tropical Diseases, London School of Hygiene and Tropical Medicine, London, UK; 2 Mahidol Oxford Tropical Medicine Research Unit, Faculty of Tropical Medicine, Mahidol University, Bangkok, Thailand; 3 Centre for Tropical Medicine and Global Health, Nuffield Department of Clinical Medicine, University of Oxford, Oxford, UK; 4 Faculty of Health, Medicine and Life Sciences, Maastricht University, Maastricht, The Netherlands; 5 Centre for Social Sciences and Global Health, University of Amsterdam, Amsterdam, The Netherlands; 6 Amsterdam Institute for Global Health and Development, Amsterdam, The Netherlands

**Keywords:** Malaria, Forest, At-risk-groups, Greater Mekong Sub-region, Qualitative research, Social science, Interventions, ITNs, Prophylaxis, MDA, Mass screening and treatment

## Abstract

**Background:**

Despite decreases in incidence and related mortality, malaria remains a major public health challenge in the Greater Mekong Sub-region (GMS). The emergence of artemisinin resistance threatens these gains and has prompted efforts to accelerate elimination in the region. In the GMS, transmission now clusters in hotspots along international borders and among high-risk populations, including forest-goers. To eliminate malaria in the region, interventions must target such hard-to-reach populations. This review provides a comprehensive overview of the qualitative research on behaviours and perceptions that influence uptake of and adherence to malaria interventions among forest-goers in the GMS.

**Methods:**

A systematic search strategy was used to identify relevant sources, including database (OVID SP, PubMed, ISI Web of Knowledge) and bibliographic searches. Relevant findings from qualitative research methods were extracted and thematic analysis undertaken.

**Results:**

Of 268 sources retrieved in searches twenty-two were reviewed. Most reported studies were conducted in Cambodia (n = 10), and were published after 2014 (n = 16). Four major themes emerged that are particularly relevant to the design of intervention packages targeted at forest-goers: (1) understanding of malaria and perceived risk; (2) preventive measures used when visiting the forest; (3) behaviours that put forest-goers at risk of infection; and, (4) malaria-related treatment seeking. There were notable differences across the reviewed articles that suggest the need for a locally tailored approach.

**Conclusion:**

A more detailed characterization of forest activities is needed but research on this topic raises methodological challenges. Current vector control measures have limitations, with use of insecticidal-treated nets, hammocks and repellents influenced by the type of forest activities and the characteristics of these measures. In contrast, anti-malarial drugs, for example, as chemoprophylaxis, hold promise but require further evaluation.

**Electronic supplementary material:**

The online version of this article (10.1186/s12936-019-2666-5) contains supplementary material, which is available to authorized users.

## Background

Despite a steady decrease in malaria incidence and malaria-related mortality, the disease remains an important health and socioeconomic burden in the Greater Mekong Sub-region (GMS) [1]. Progress has been spurred by increased funding, improved vector control, enhanced case detection and the availability of effective anti-malarial treatments [2]. Concurrently, selection pressure on parasite populations in the region has resulted in the emergence of artemisinin resistance [3–5]. With no alternative anti-malarials available to replace artemisinin-derived compounds as frontline treatment, a public health crisis could ensue if resistant parasites spread to Africa [6, 7].

In response to this growing threat, health ministries of the GMS have set themselves the goal of malaria elimination by 2030 [8, 9]. As the region has progressed towards elimination, remaining malaria parasite reservoirs have clustered along international borders and forested areas [10–12]. In these areas, malaria remains endemic in high-risk populations, including mobile migrant workers and forest-goers [13, 14]. Poor adherence to measures which prevent contact with vectors, e.g. insecticide-treated bed nets (ITNs), long-lasting insecticidal hammocks (LLIHs) and mosquito repellents [15], puts them at an increased risk of acquiring malaria [14].

Intervention packages that specifically target at-risk groups are necessary to accelerate malaria elimination. The effectiveness of interventions targeted at forest-goers (and any group) depends upon their uptake and adherence. Understanding attitudes towards current interventions and malaria-related behaviours is key to the appropriate design of future strategies to maximise their impact. Social science research that employs qualitative methods, such as interviews, focus group discussion and observations, can offer insights into the factors that influence behaviours and perceptions that influence uptake and adherence.

This review provides a comprehensive overview of the qualitative research conducted on forest-goers in the GMS since the turn of the Century. The article examines behaviours and perceptions that influence uptake of and adherence to malaria interventions with a view to informing the design of appropriately tailored intervention packages. Due to the relative paucity of qualitative research on this topic, this article identifies gaps in the current evidence to guide future studies.

## Methods

### Literature search

To identify relevant articles, database searches were carried out in OVID SP, PubMed and ISI Web of Knowledge in June 2018. Test searches were used to refine the appropriate terms and ensure that relevant studies were identified. The final search terms are shown in Table 1. These search terms were chosen to ensure that the sources identified were specific to the region of interest, based on research using qualitative social science research methods (including ethnographic methods, as used in previous systematic reviews of qualitative research [16]) and all research that mentioned forest-related activities or -goers and malaria. Grey literature was identified using Google, Social Science Research Network, OpenGrey and EThOS.

**Table 1:** Search terms

	OVID SP, PubMed and Web of Knowledge		Google, Social Science Research Network, OpenGrey and EThOS
	Malaria		Malaria
AND	Greater Mekong Sub-region OR GMS OR Burma OR Myanmar OR Thailand OR Vietnam OR Cambodia OR China OR Yunnan OR Guangxi Zhuang OR Lao*	AND	GMS
AND	Forest	AND	Forest
AND	Qualitative OR interview OR discussion OR sociology* OR perception* OR belief* OR attitude*	AND	Qualitative

The searches identified 268 articles, which were reduced to 141 after removing duplicates. Titles and abstracts of the articles were downloaded into Mendeley and were screened according to the following inclusion criteria: original research related to malaria; conducted in the GMS; written in English; utilizing qualitative methods including interviews, group discussion and observations.

**Fig. 1:**
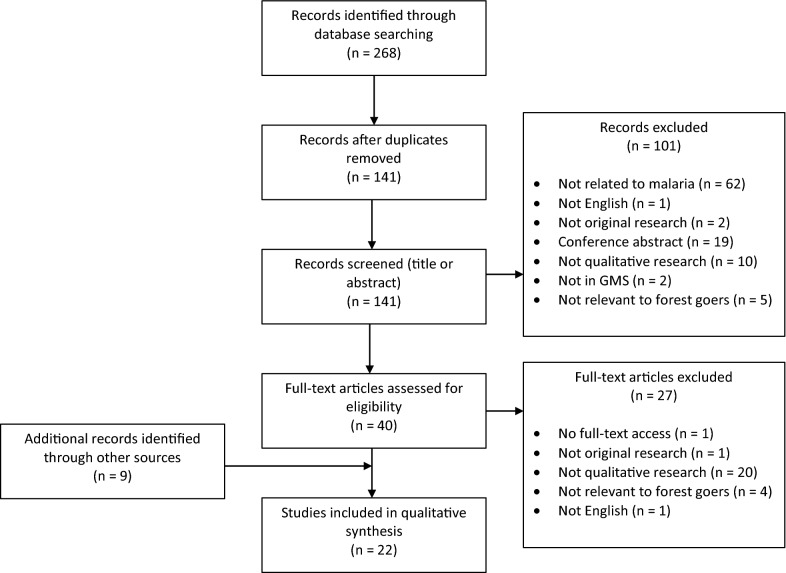
PRISMA flow diagram

The full texts of 40 articles were retrieved, read and 13 articles were selected as meeting inclusion criteria. A hand search of the bibliographies of the selected articles was also conducted, along with a Google Scholar search, identifying a further nine articles (see Fig. 1 for an overview of the selection process). A total of 22 articles were included for thematic analysis of qualitative findings. For articles that used both qualitative and quantitative methods, only findings derived from qualitative methods were considered. Information on the data collection methods, date published, study location and target groups were also extracted for each article.

### Data analysis

A deductive and inductive approach was used to analyse the data from selected articles. Using the deductive approach, specific data for pre-set themes were explored and during the process of review emerging themes were developed using the inductive approach [17]. Themes relating to factors that may influence malaria-related interventions targeting forest-goers were identified and relevant data were extracted and categorized based on the thematic content. The data within the themes were explored for patterns and interpretation based on the context where the studies took place. The relevant themes and the extracted data are presented below. One of the co-authors (SN) extracted the data from all sources and another co-author (BA) undertook a second round of data extraction for a sub-set of sources as a means of checking and ensuring that no relevant information was overlooked.

## Results

### Study characteristics

Of the 22 studies reviewed, the majority were conducted in Cambodia (n = 10), followed by Vietnam (n = 4), Myanmar (n = 3), Thailand (n = 2), Laos (n = 1), Thai-Burmese border (n = 1), and Vietnamese-Cambodian border (n = 1) (Table 2). Articles were published between 1986 and 2018, with the majority published after 2014 (16/22; 72%) illustrating a growing trend in qualitative studies in recent years. The studies utilized various data collection techniques, including individual interviews (n = 21), participant observations (n = 17), and in-depth group discussions (n = 10). Of the 22 studies, 13 employed a mixed-methods approach combining qualitative and quantitative methods, such as questionnaire-based surveys and focus group discussions. In most studies, forest-goers were not the primary focus, and instead were among other respondents which included community leaders, health providers and malaria patients.

**Table 2:** Characteristics of the reviewed articles

First author	Year	Country	Duration of data collection	Qualitative methods		
				Focus groups	Individual interviews	Observations
Adhikari	2018	Laos	11 months (Sep 2015 to Aug 2016)	12 FGDs with 100 participants	31	Yes
Bannister-Tyrrell	2018	Vietnam	2 months (April to June 2016)	No	22 in-depth interview, 10 informal conversations	Yes
Chen	2017	Vietnam	2 months (Dec 2015 to Jan 2016)		61	
Crawshaw	2017	Myanmar	Unspecified	42	5	
Grietens	2010	Vietnam	3 months (July 2005 to September 2006)		101	Participated in everyday activities inc. forest activities
Grietens	2012	Vietnam	3 months (between July 2005 and September 2006)	Yes	Semi-structured	Ethnography
Grietens	2015	Cambodia	Unspecified (during 2012)		Yes	Yes
Gryseels	2013	Cambodia	4 months (April to July 2010)		126	Yes
Gryseels	2015a	Cambodia	Between 2012 and 2013		320 individual interviews and 759 informal conversations	Smelt household member’s arms for repellent
Gryseels	2015b	Cambodia/Vietnam Border	5 months (between 2008 and 2010)		257	Observed daily life
Gryseels	2015c	Cambodia	Unspecified (during 2012)		153	Yes
Lim	2017	Cambodia	3 days in each village (20)	1	18	Yes
Liverani	2017	Cambodia	2013 duration unspecified		71	
Lyttleton	2016	Thailand	2 weeks in November 2014		Yes	Observed daily life, health outreach activity
Panvisavas	2001	Thai Myanmar Border	8 months in 1999	5	Yes	
Pell	2017	Cambodia	2015 and 2016	Yes	40	Yes
Sahan	2017	Myanmar	4 months (March to July 2015)		45	Yes
Shafique	2016	Cambodia	8 months (August 2010 to March 2011)	6	13	Yes
Singhanetra-Renard	1986	Thailand	June 1985 to March 1986		Yes	Yes
Taffon	2018	Cambodia	4 months (December 2015 to March 2016)	86	9	Yes
Verschuere	2017	Cambodia	4 months (August to November 2013)	7 (49 participants in total)	42	Observed daily life
Wharton-Smith	2014	Myanmar	2 months (May to June 2014)	Yes	Yes	

### Qualitative synthesis of findings

During the systematic analysis of the articles, four major themes emerged that are particularly relevant to the design of intervention packages specifically targeted towards forest-goers (Additional file 1) (1) forest-goers’ understanding of malaria and their perceived risk; (2) their malaria-related treatment seeking behaviour; (3) the preventative measures used when visiting the forest; and, (4) the behaviours that put them at risk of infection.

### Understanding of malaria

In most of the reviewed sources, respondents demonstrated a basic understanding of malaria and its symptoms. However, in one study along the Thai/Burmese border, malaria was viewed as a symptom rather than a disease [18]. Mosquito bites were often described as the cause of malaria, and misconceptions regarding the mode of transmission were common: malaria was linked to drinking or bathing in contaminated water, exposure to contaminated wind, tiredness, ill health, poor hygiene, or eating specific foods. In Cambodia, supernatural deities, ghosts, sorcery and forest spirits were reported as causes of malaria [19–23].

Study respondents commonly associated visits to the forest with increased risk of contracting malaria [22–27] and, in Myanmar, malaria was even referred to as forest-sickness [18]. Despite risking malaria infection, respondents described that visiting forests was essential for sustenance, usually from swidden farming, hunting or logging. In some settings, malaria was perceived as an insignificant risk because mosquitoes in the forest were not seen as malaria vectors [28] or because only unhealthy individuals could become infected [29]. In one article, the ease with which malaria could be treated also reduced the perceived danger of malaria infection [25]. Study respondents referred to fever and chills as the main symptoms of malaria [18, 21]. However, understanding of asymptomatic malaria was discussed in only one article [24].

### Prevention and control interventions

The type of preventative measures used by respondents to counter malaria infection depended on several factors, including availability, durability, cost and practicalities of use. In some studies, the irritation experienced from mosquito bites, rather than the fear of contracting malaria, prompted the use of protective measures, such as wearing long-sleeved clothing [30, 31].

#### Smoke, mosquito coils and long sleeves

Wearing long-sleeved/legged clothing to prevent mosquito bites while working in the forest was reported in several studies [20–22, 24, 26, 27, 32, 33]. However, due to the strenuous nature of their work, forest-goers would often take off long-sleeved/legged clothing if they felt too hot [25, 27, 32]. Practices, such as burning leaves and mosquito coils [21, 22, 25–27, 31–34] were also popular ways of deterring mosquitoes while in the forest and were used, either in conjunction with or to substitute other malaria prevention methods. In Myanmar, for instance, mosquito coils were worn in headbands and waistbands during night-time work [32]. Although practical and convenient, respondents recognized that burning fires did not provide sufficient protection, leaving them susceptible to malaria infection [25, 26] and that smoke inhalation could have further negative impacts on their health [27].

#### Repellents

Mosquito repellents were not commonly used among forest-goers. Although some respondents described mosquito repellents as a useful way to prevent mosquito bites when bed nets could not be used or to get rid of other insects, such as lice [35], others reported that repellents were not effective [25, 27]. Because of the strong smell of the repellents, respondents in three studies described repellents as toxic and harmful to their skin [27, 32, 35]. The high cost of the repellents also presented a barrier that limited its use among respondents [27].

#### Bed nets

Bed nets were mentioned as malaria prevention tools in several studies [15, 20–22, 24, 26–30, 33, 36], but were not always used consistently or appropriately. Forest-goers would not use bed nets for reasons related to the hot and humid environment of the forest within which they stayed [15, 18, 27] and would sometimes use them as pillows or blankets [15, 27]. Some forest-goers described how it was inconvenient to carry bed nets with them, and prioritized other essential items [15, 25, 27]. In one study, bed nets were only taken to the forest if respondents spent longer than two nights there [27]. Respondents in four studies described how bed nets were left in the village for other household members to use [15, 29, 34, 36] because they did not own extra nets that could be taken to the forest. Insufficient access to bed nets was an issue reported in several sources [15, 27, 29, 34, 36]. Although free bed net distribution campaigns have taken place across the GMS, forest-goers were sometimes absent during the distribution [27] or did not have a registration card [34] and were not eligible for a free bed net. Some respondents could not afford to purchase bed nets or replace them once they became damaged [15, 27, 34].

Several studies described a preference for non-impregnated nets, usually purchased from the market, over ITNs. In one instance, these concerns were related to the perceived safety of the insecticide used in ITNs [27]. Many respondents reported that small insects could still penetrate ITNs despite the insecticide treatment, and therefore preferred market nets with smaller mesh sizes than ITNs [27, 33, 36]. Market-bought nets were also perceived as softer, making them easier to pack when going to the forest [27] and came in a variety of sizes. This was important for respondents because they preferred using larger bed nets in the village to allow the entire family to sleep together, and smaller bed nets for individual use in the forests [33].

#### Hammock nets

Although forest-goers considered hammock nets more practical than bed nets [27], they were not commonly mentioned as a malaria prevention tool because they were either unheard of [29], difficult to find [29], expensive or uncomfortable in the hot and humid environment of the forest [27]. For these reasons, respondents did not consistently use hammock nets while in the forest [15, 22, 29].

#### Mass screening and treatment, and mass drug administration

Concerns regarding the safety of blood tests affected participation in screening campaigns [20, 21], whereas, in another study, respondents avoided tests due to the fear of testing positive for narcotics or malaria, both of which would imply illegal forest work [25]. Enrolment in mass-drug administration (MDA) was also affected, mainly by concerns regarding the safety of artemisinin-based combination therapy (ACT) as a result of real and perceived side-effects experienced after taking them [21] and by apprehension about blood tests [23]. In Laos, for instance, the fear of needles and losing too much blood were common worries among potential MDA participants [23].

### Risky behaviour

Several behaviours that place forest-goers at increased risk of malaria were identified. Socializing during the evenings and delayed sleeping times led to inconsistent bed net usage and increased exposure to mosquito vectors [30, 33]. In one study, a respondent was aware of this risk and would therefore spend leisure time under a bed net to avoid mosquito bites [27]. A common misconception among respondents was that alcohol consumption provided protection against mosquito bites and thus malaria [27, 29, 34]. Blankets were perceived to provide sufficient protection against mosquito bites while sleeping [27, 31, 34, 37] leading to reduced bed net usage. Respondents in one study reported being bitten by mosquitoes when urinating and defecating at night or in the early morning due to the lack of mosquito-proof latrines in the forest [33]. The illegal nature of some of the activities carried out in the forest resulted in some respondents resorting to night-time work when mosquito vector densities are higher [25, 31].

Many forest-goers missed participating in malaria prevention interventions such as MDA [18, 21, 24], active case detection [26], bed net distributions [27], and the dissemination of health information [22, 27, 29, 34]. Activities that promoted appropriate prevention practices through village drama projects [20] and positive deviance, a method that encourages preventive behaviours already found in the community [22], were well received, with respondents reporting behavioural changes and increased uptake of malaria prevention methods. Some respondents therefore requested that they be informed prior to the start of these activities so that they could arrange being back in the village in order to attend [21] or requested that these activities take place more often [27].

### Treatment-seeking behaviours

With diverse malaria treatment outlets available, treatment seeking was highly heterogeneous among forest-goers and often involved multiple points of care. Treatment choices were influenced by socio-economic factors, local medical traditions, accessibility and quality of service.

Traditional medicine and healing practices such as coining (a traditional dermabrasion therapy used to relieve fevers), fanning and fever baths were commonly reported as ways of alleviating malaria symptoms [18, 19, 22, 23, 34, 38]. In some cases, traditional medicine was used before seeking biomedical treatment in the public or private sector [18, 22, 38]. For others, traditional medicine was a last resort if symptoms did not improve after taking anti-malarials [19, 23]. The perceived cause of the disease also played a role in respondents’ choice of treatment: they were more likely to resort to traditional medicine when they suspected malaria to have a supernatural cause [19].

Self-treatment (using drugs purchased from pharmacies, groceries and mobile vendors) was frequently reported [22, 23, 29, 38]. Respondents in two studies described taking drugs with them to the forest in case they fell ill [19, 29]. A variety of treatment options were available from drug outlets, including artemisinin-based combination therapy (ACT), artemether injections and drug cocktails which consisted of antibiotics, anti-pyretics, artemisinin monotherapies or chloroquine [19]. In one study, drug cocktails and artemether injections were the preferred choice of treatment because they were considered to be more effective, offered faster relief and had milder side effects when compared to ACTs [19].

In several articles, study respondents failed to adhere to the full course of treatment because they could either not afford it [19] or would terminate treatment once symptoms had resolved [25, 29]. Although the preference for self-treatment often stemmed from convenience and ability to avoid undesired drugs, such as the ACT artesunate-mefloquine, which was perceived to be associated with severe side effects, one Cambodian study demonstrated that health-seeking patterns also depended on the type of malaria diagnosis, with respondents more likely to self-treat if diagnosed with *Plasmodium vivax* compared to *Plasmodium falciparum* [23].

Across the GMS, public health facilities provide malaria diagnosis and treatment free of charge. Nevertheless, there was a general preference for the private sector among respondents. This was attributed to the poor accessibility of public health facilities [22, 24] and long waiting times [23]. Respondents also preferred the private sector for its superior customer service and flexible opening hours [19, 23, 29, 38]. In certain settings, such as in Vietnam and Thailand, a national identity card was a requirement for accessing free services provided at health centres [23, 29] and as a result, many migrants (mostly from neighbouring countries) did not have free access and therefore frequently sought cheaper treatment in the private sector. In one study, respondents were deterred by hidden costs, such as consultation fees, that had to be paid in the public sector [29].

In four articles, community health workers (CHWs), who support public health coverage in remote and rural villages, were described as the first point of treatment once malaria was suspected [19, 21, 23, 24]. Nevertheless, CHWs were viewed as unreliable [19, 22, 38] because they were either unavailable [38], unwilling to visit patients who lived far away [38], or ran out of rapid diagnostic tests (RDTs) and treatment [19]. Some respondents were also disgruntled by CHWs’ limited capacity to treat only malaria [22, 38] and would therefore seek diagnosis and treatment from alternative health providers if they were unsure whether they had malaria or if they suspected secondary illnesses.

## Discussion

The findings provide an overview of qualitative research on behaviours and perceptions that influence uptake of and adherence to malaria prevention and control interventions among forest-goers in the GMS. The review focused on this group because they are deemed the priority population for the region’s malaria elimination [13]: forest-goers are at particular risk of sub-clinical malaria infections [39] and report sub-optimal use of preventative interventions, particularly ITNs [14]. As a result of the greater infection outside villages, forest-goers are at particular risk of *Plasmodium vivax.* Because they come into contact with macaques and other monkey species carrying predominantly non-human *Plasmodium* species, e.g. *Plasmodium cynomolgi* and *Plasmodium knowlesi*, it is increasingly recognized that forest-goers are at risk of zoonotic malaria [40].

Four major themes are particularly relevant to the design of intervention packages specifically targeted towards forest-goers: (1) the understanding of malaria and perceived risk; (2) the preventative measures used when visiting the forest ; (3)  specific behaviours that put forest-goers at risk of infection; and, (4) malaria-related treatment seeking behaviours. There were notable differences among these themes across the reviewed articles with many context-specific issues. This suggests the need for a tailored approach when designing intervention for forest-goers.

### Vector control interventions

As in other regions, interventions that target malaria vectors have been widely promoted in the GMS [41]. Although respondents in some of the reviewed studies reported using recommended measures, several limitations for forest-goers were highlighted.

If forest-goers took protective measures, it was often because of the nuisance of mosquito bites, not to prevent malaria [30, 31]. Although they generally associated malaria with mosquitoes, mosquito bites and spending time in forested areas [22–27] as highlighted elsewhere [e.g. 42], study respondents linked a range of other aetiological factors to malaria, including a lack of cleanliness and supernatural forces [19–23]. Forest-goers’ concern about malaria infection also varied across the settings [28].

Respondents described rudimentary protection measures, such as wearing long-sleeved shirts and long trousers when in forested areas [20–22, 24, 26, 27, 32, 33]. However, due to the strenuous nature of forest work, this was often impractical [25, 27, 32]. Burning leaves to repel mosquitoes was popular but recognized as inadequate [21, 25–27, 31, 33, 34] and potentially harmful [27]. Mosquito coils were occasionally mentioned [22, 27, 32]. The, strong smell of repellents [27, 32, 35] and their high cost [27] were reasons that they were not readily used.

The use of hammocks and ITNs was influenced by their cost and characteristics, e.g., the size of the mesh, and whether they were compatible with users’ forest shelters and sleeping areas [27, 33, 36]. Insecticide-treated hammocks [15, 22, 29] or ITNs [15, 25, 27] were readily abandoned or irrelevant because of the nature of activities in forested areas. Evening socializing and alcohol consumption along with other night-time activities, such as urinating, defecating [33], logging or hunting (in an attempt to keep their illegal activities clandestine) [25, 31] meant that some forest-goers were particularly exposed to mosquito vectors [27, 29–31, 33, 34, 37]. These factors are further compounded by the biting behaviour of the exophagic forest dwelling vectors, *Anopheles dirus* and *Anopheles minimus* [43].

A more human-centred approach to the design of vector control interventions, whereby end-user preferences and practices are incorporated into a collaborative process of product development [44] might promote more optimum use. Such an approach is increasingly popular in the development of a range of health-related interventions [e.g. 45]. Forest-goers’ limited access to ITNs results from their absence during distribution campaigns that target villages, and the cost of ITNs. These barriers could be mitigated by careful planning of the distribution, based upon an understanding of forest-going patterns.

Across the reviewed studies, relatively little was reported about the specifics of forest-going, particularly in terms of locations and practices. This is because the articles rarely focused on this sub-group or because of the sometimes illegal nature of forest activities [25]. Therefore, although a more detailed characterization of forest activities could help to understand where and when they encounter the relevant vectors (and, for example, tailor distribution of ITNs or hammocks), this is likely to raise methodological, ethical and legal issues.

### Mass drug administration, mass screening and treatment, and prophylaxis

Several proposed interventions that aim to accelerate malaria elimination (in the GMS) target the asymptomatic reservoir of infections (e.g. MDA, mass screening and treatment using ultra high-sensitive diagnostics). These approaches along with the prophylactic use of anti-malarials among at-risk groups entail ingesting a pharmaceutical drug when individuals are not experiencing symptoms. Few studies however addressed the topic of asymptomatic malaria and the attitudes towards taking anti-malarials when asymptomatic [21].

Given the importance of asymptomatic malaria for continued transmission in low-transmission, pre-elimination settings [46], effective active case detection programmes must include highly sensitive diagnostic techniques [47]. However, mass- or targeted screening with sufficiently sensitive diagnostics (e.g. using laboratory methods, such as high-volume ultra-sensitive quantitative PCR) are currently not feasible due to the cost and delay between sampling and the result [47]; furthermore, although promising, point-of-care, ultra-sensitive RDTs still require further clinical investigation [48].

Alongside pilot studies across the GMS, a programme of mixed-methods research has addressed attitudes to malaria and experiences of MDA for malaria elimination [18, 21, 24, 49, 50]. The results of these studies indicated that people participated in MDA because they were familiar with and were concerned about malaria, also because they were aware of MDA and its aim of eliminating malaria. A high uptake was prompted, at least in part, by an extensive programme of community engagement that addressed community members’ concerns about side effects of anti-malarials [51, 52]. The high-uptake suggests that MDA could be a promising intervention in high-risk populations even though forest workers may only benefit indirectly.

The reviewed studies suggest that forest-goers’ periodic absence from settlements meant that they were often not included in malaria prevention programmes [18, 21, 24, 26]. Familiarity with the timing of forest activities (or targeting forest locations) is therefore crucial. These programmes must also address the reported concerns about the safety of the blood tests [20, 21], or fears that the test results would be used to infer illegal (forest) activities [25].

Among the reviewed articles, there were reports that some people carry anti-malarial drugs with them to the forest in case they fall ill [19, 29]. Respondents used a variety of treatment options, including potentially harmful drug cocktails of antibiotics, antipyretics and anti-malarials [19]. The therapy was often obtained from private providers even though, across the GMS, public health facilities provide malaria diagnosis and treatment free of charge [22–24]. In part, this resulted from a lack of confidence in CHWs charged with diagnosis [19, 21–24, 38]. Poor adherence to prescribed treatment due to the costs [19] or because of complacency after the symptoms subsided [25, 29] was also reported.

A willingness to carry and ingest anti-malarials whilst visiting forests suggests that the impact of providing of prophylactic drugs to forest-goers should be examined. A recent pilot study among Vietnamese forest rangers indicates potential for such an approach [53]. In addition to its impact on clinical outcomes and parasitaemia, any evaluation must address questions of feasibility, given the reported poor adherence to therapeutic courses of anti-malarials. Strengthening the CHW network to overcome limitations that were described in the reviewed studies (difficulties in finding CHWs, lack of RDTs and anti-malarials, and the fact that they are unable to treat other illnesses [19, 22, 38]) could facilitate implementation and improve sustainability.

Their readiness to self-administer anti-malarials in the forest indicates that providing forest-goers with RDTs for self-testing alongside an ACT for self-treatment after a positive RDT result might be acceptable. This approach could potentially speed appropriate treatment for clinical malaria cases in areas where access to primary health services is limited by distance or terrain, as is the case in many forested zones of the GMS. Such a strategy has been piloted amongst international travellers who visit malaria endemic areas and requires some preparatory training to promote optimal use [54]. Although this strategy might improve the management of individual clinical cases, it is unlikely to address the contribution of asymptomatic infections to transmission in this region and therefore has limitation in terms of contributing to the regional elimination of malaria [55].

### Strengths and limitations

This is the first article to review the qualitative social science research on the malaria-related perceptions and behaviours of forest-goers in the GMS. The findings are limited by the fact that forest-goers were not the central respondent group in all studies, although in all included studies forest-goers were interviewed, or their behaviour discussed. Not all countries in the GMS were included, because the malaria burden is not equally distributed across the region; the included sources described studies conducted in the countries with the highest malaria burden. Unavailable texts were generally older articles (before 2000). Because new initiatives and interventions have been introduced over the last 20 years, unavailable articles may no longer be representative of the current malaria landscape, and the absence of these sources is unlikely to affect the main findings.

## Conclusion

This review provides a comprehensive overview of qualitative social science research that has examined behaviours and perceptions that influence uptake of and adherence to malaria prevention and control interventions among forest-goers in the GMS. Because they are at particular risk of sub-clinical malaria infections, which fosters continued transmission, forest-goers are a priority population for the region’s malaria elimination programmes. The findings highlight the limitations of vector control measures for this population group, with use of ITNs, hammocks and repellents influenced by the type of forest activities and the characteristics of these measures. A human-centred approach to the design of ITNs and hammocks offers potential to overcome some of these challenges. The findings also indicate that delivering anti-malarial drugs to this population group (as mass screening and treatment, MDA or prophylaxis) is potentially complex: forest-goers are often absent from village-based interventions, express concerns about blood tests and their adherence to treatment courses is sometimes sub-optimal. Research on the timing and location of forest activities is needed to optimize the delivery of interventions but, considering the sometimes illegal nature of forest activities, this brings methodological and ethical challenges. Operational research alongside the clinical evaluation of prophylaxis and self-testing and treatment for this population group is needed.

## Additional file

**Additional file 1.** Summary of data extraction by theme.

## References

[1] WHO. Strategy for malaria elimination in the Greater Mekong subregion: 2015–2030. Geneva: World Health Organization. http://www.wpro.who.int/mvp/documents/en/. http://www.wpro.who.int/mvp/documents/en/. Accessed 18 Aug 2016.

[2] Okayas H (2018). Mekong Malaria Elimination Programme.

[3] Noedl H, Se Y, Schaecher K, Smith BL, Socheat D, Fukuda MM (2008). Evidence of artemisinin-resistant malaria in Western Cambodia. N Engl J Med.

[4] Dondorp AM, Nosten F, Yi P, Das D, Hanpithakpong W, Lee SJ (2009). Artemisinin resistance in *Plasmodium falciparum* malaria. N Engl J Med.

[5] Ashley EA, Dhorda M, Fairhurst RM, Amaratunga C, Lim P, Suon S (2014). Spread of artemisinin resistance in *Plasmodium falciparum* malaria. N Engl J Med.

[6] Wootton JC, Feng X, Ferdig MT, Cooper RA, Mu J, Baruch DI (2002). Genetic diversity and chloroquine selective sweeps in *Plasmodium falciparum*. Nature.

[7] Roper C, Pearce R, Nair S, Sharp B, Nosten F, Anderson T (2004). Intercontinental spread of pyrimethamine-resistant malaria. Science.

[8] WHO. Eliminating malaria in the Greater Mekong Subregion. World Health Organ. 2010.

[9] WHO (2018). World Malaria Report 2018.

[10] Imwong M, Nguyen TN, Tripura R, Peto TJ, Lee SJ, Lwin KM (2015). The epidemiology of subclinical malaria infections in South-East Asia: findings from cross-sectional surveys in Thailand-Myanmar border areas, Cambodia, and Vietnam. Malar J..

[11] Tripura R, Peto TJ, Veugen CC, Nguon C, Davoeung C, James N (2017). Submicroscopic Plasmodium prevalence in relation to malaria incidence in 20 villages in western Cambodia. Malar J..

[12] Tripura R, Peto TJ, Chalk J, Lee SJ, Sirithiranont P, Nguon C (2016). Persistent *Plasmodium falciparum* and *Plasmodium vivax* infections in a western Cambodian population: implications for prevention, treatment and elimination strategies. Malar J..

[13] Guyant P, Canavati SE, Chea N, Ly P, Whittaker MA, Roca-Feltrer A (2015). Malaria and the mobile and migrant population in Cambodia: a population movement framework to inform strategies for malaria control and elimination. Malar J..

[14] Regional Office for South-East Asia, World Health Organization. Approaches for mobile and migrant populations in the context of malaria multi-drug resistance and malaria elimination in the Greater Mekong Subregion. New Delhi, India: WHO Regional Office for South-East Asia; 2016. http://www.who.int/iris/handle/10665/204351. Accessed 9 Aug 2018.

[15] Grietens KP, Xuan XN, Van Bortel W, Duc TN, Ribera JM, Nhat TB (2010). Low perception of malaria risk among the Ra-glai ethnic minority in south-central Vietnam: implications for forest malaria control. Malar J..

[16] Pell C, Straus L, Andrew EVW, Menaca A, Pool R (2011). Social and cultural factors affecting uptake of interventions for malaria in pregnancy in Africa: a systematic review of the qualitative research. PLoS ONE.

[17] Fereday J, Muir-Cochrane E (2006). Demonstrating rigor using thematic analysis: a hybrid approach of inductive and deductive coding and theme development. Int J Qual Methods..

[18] Sahan K, Pell C, Smithuis F, Phyo AK, Maung SM, Indrasuta C (2017). Community engagement and the social context of targeted malaria treatment: a qualitative study in Kayin (Karen) State, Myanmar. Malar J..

[19] Gryseels C, Uk S, Erhart A, Gerrets R, Sluydts V, Durnez L (2013). Injections, cocktails and diviners: therapeutic flexibility in the context of malaria elimination and drug resistance in Northeast Cambodia. PLoS ONE.

[20] Lim R, Tripura R, Peto TJ, Sareth M, Sanann N, Davoeung C (2017). Drama as a community engagement strategy for malaria in rural Cambodia. Wellcome Open Res.

[21] Pell C, Tripura R, Nguon C, Cheah P, Davoeung C, Heng C (2017). Mass anti-malarial administration in western Cambodia: a qualitative study of factors affecting coverage. Malar J..

[22] Shafique M, Edwards HM, De Beyl CZ, Thavrin BK, Min M, Roca-Feltrer A (2016). Positive deviance as a novel tool in malaria control and elimination: methodology, qualitative assessment and future potential. Malar J..

[23] Verschuere J, Decroo T, Lim D, Kindermans JM, Nguon C, Huy R (2017). Local constraints to access appropriate malaria treatment in the context of parasite resistance in Cambodia: a qualitative study. Malar J..

[24] Adhikari B, Phommasone K, Kommarasy P, Soundala X, Souvanthong P, Pongvongsa T (2018). Why do people participate in mass anti-malarial administration? Findings from a qualitative study in Nong District, Savannakhet Province, Lao PDR (Laos). Malar J..

[25] Lyttleton C (2016). Deviance and resistance: malaria elimination in the greater Mekong subregion. Soc Sci Med.

[26] Taffon P, Rossi G, Kindermans JM, Van den Bergh R, Nguon C, Debackere M (2018). ‘I could not join because I had to work for pay.’: a qualitative evaluation of falciparum malaria pro-active case detection in three rural Cambodian villages. PLoS ONE..

[27] Wharton-Smith A, Shafique M (2014). A qualitative study to assess consumer preferences and barriers to use of long lasting insecticidal nets in Myanmar.

[28] Grietens KP, Gryseels C, Dierickx S, Bannister-Tyrrell M, Trienekens S, Uk S (2015). Characterizing types of human mobility to inform differential and targeted malaria elimination strategies in Northeast Cambodia. Sci Rep..

[29] Chen I, Thanh HNT, Lover A, Thao PT, Luu TV, Thang HN (2017). Malaria risk factors and care-seeking behaviour within the private sector among high-risk populations in Vietnam: a qualitative study. Malar J..

[30] Bannister-Tyrrell M, Xa NX, Kattenberg JH, Van Van N, Dung VKA, Hieu TM (2018). Micro-epidemiology of malaria in an elimination setting in Central Vietnam. Malar J..

[31] Singhanetra-Renard A (1986). Population movement, socio-economic behaviour and the transmission of malaria in northern Thailand. Southeast Asian J Trop Med Public Health.

[32] Crawshaw AF, Maung TM, Shafique M, Sint N, Nicholas S, Li MS (2017). Acceptability of insecticide-treated clothing for malaria prevention among migrant rubber tappers in Myanmar: a cluster-randomized non-inferiority crossover trial. Malar J..

[33] Gryseels C, Durnez L, Gerrets R, Uk S, Suon S, Set S (2015). Re-imagining malaria: heterogeneity of human and mosquito behaviour in relation to residual malaria transmission in Cambodia. Malar J..

[34] Panvisavas S (2001). Poverty and malaria: a study in a Thai-Myanmar border area. Southeast Asian J Trop Med Public Health.

[35] Gryseels C, Uk S, Sluydts V, Durnez L, Phoeuk P, Suon S (2015). Factors influencing the use of topical repellents: implications for the effectiveness of malaria elimination strategies. Sci Rep..

[36] Gryseels C, Grietens KP, Dierickx S, Xuan XN, Uk S, Bannister-Tyrrell M (2015). High mobility and low use of malaria preventive measures among the Jarai male youth along the Cambodia-Vietnam Border. Am J Trop Med Hyg.

[37] Grietens KP, Xuan XN, Ribera JM, Duc TN, van Bortel W, Ba NT (2012). Social Determinants of long lasting insecticidal hammock-use among the Ra-Glai ethnic minority in Vietnam: implications for forest malaria control. PLoS ONE.

[38] Liverani M, Nguon C, Sok R, Kim D, Nou P, Nguon S (2017). Improving access to health care amongst vulnerable populations: a qualitative study of village malaria workers in Kampot, Cambodia. BMC Health Serv Res..

[39] Parker DM, Tripura R, Peto TJ, Maude RJ, Nguon C, Chalk J (2017). A multi-level spatial analysis of clinical malaria and subclinical Plasmodium infections in Pailin Province, Cambodia. Heliyon..

[40] Imwong M, Madmanee W, Suwannasin K, Kunasol C, Peto TJ, Tripura R, et al. Asymptomatic natural human infections with the simian malaria parasites *Plasmodium cynomolgi* and *Plasmodium knowlesi*. J Infect Dis. 2018. 10.1093/infdis/jiy519. (Epub ahead of print). 10.1093/infdis/jiy519 PMC6376906 30295822

[41] WHO (2017). Achieving and maintaining universal coverage with long-lasting insecticidal nets for malaria control.

[42] Menaca A, Pell C, Manda-Taylor L, Chatio S, Afrah NA, Were F (2013). Local illness concepts and their relevance for the prevention and control of malaria during pregnancy in Ghana, Kenya and Malawi: findings from a comparative qualitative study. Malar J..

[43] Trung HD, Van Bortel W, Sochantha T, Keokenchanh K, Briët OJT, Coosemans M (2005). Behavioural heterogeneity of Anopheles species in ecologically different localities in Southeast Asia: a challenge for vector control. Trop Med Int Health..

[44] Brown T, Wyatt J (2010). Design thinking for social innovation. Dev Outreach World Bank.

[45] Mullaney T, Pettersson H, Nyholm T, Stolterman E (2012). Thinking beyond the cure: a case for human-centered design in cancer care. Int J Design..

[46] Lindblade KA, Steinhardt L, Samuels A, Kachur SP, Slutsker L (2013). The silent threat: asymptomatic parasitemia and malaria transmission. Expert Rev Anti Infect Ther..

[47] Sturrock HJW, Hsiang MS, Cohen JM, Smith DL, Greenhouse B, Bousema T (2013). Targeting asymptomatic malaria infections: active surveillance in control and elimination. PLoS Med..

[48] Landier J, Haohankhunnatham W, Das S, Konghahong K, Christensen P, Raksuansak J (2018). Operational performance of a *Plasmodium falciparum* ultrasensitive rapid diagnostic test for detection of asymptomatic infections in Eastern Myanmar. J Clin Microbiol.

[49] Adhikari B, Phommasone K, Pongvongsa T, Kommarasy P, Soundala X, Henriques G (2017). Factors associated with population coverage of targeted malaria elimination (TME) in southern Savannakhet Province, Lao PDR. Malar J..

[50] Peto TJ, Debackere M, Etienne W, Vernaeve L, Tripura R, Falq G (2018). Community participation during two mass anti-malarial administrations in Cambodia: lessons from a joint workshop. Malar J..

[51] Peto TJ, Tripura R, Davoeung C, Nguon C, Nou S, Heng C (2018). Reflections on a community engagement strategy for mass antimalarial drug administration in Cambodia. Am J Trop Med Hyg.

[52] Adhikari B, Pell C, Phommasone K, Soundala X, Kommarasy P, Pongvongsa T (2017). Elements of effective community engagement: lessons from a targeted malaria elimination study in Lao PDR (Laos). Glob Health Action..

[53] Son DH, Thuy-Nhien N, von Seidlein L, Le Phuc-Nhi T, Phu NT, Tuyen NTK (2017). The prevalence, incidence and prevention of *Plasmodium falciparum* infections in forest rangers in Bu Gia Map National Park, Binh Phuoc province, Vietnam: a pilot study. Malar J..

[54] Berthod D, Rochat J, Voumard R, Rochat L, Genton B, D’Acremont V (2017). Self-diagnosis of malaria by travellers: a cohort study on the use of malaria rapid diagnostic tests provided by a Swiss travel clinic. Malar J..

[55] von Seidlein L (2014). The failure of screening and treating as a malaria elimination strategy. PLoS Med..

