# Supplementary material for: How can interventions that target forest-goers be tailored to accelerate malaria elimination in the Greater Mekong Subregion? A systematic review of the qualitative literature
Source: Malar J. 2019 Feb 1;18:32. doi: 10.1186/s12936-019-2666-5 (PMC6359845; doi:10.1186/s12936-019-2666-5)
Supplement: Supplementary file 1 — Additional file 1. Summary of data extraction by theme. [file 12936_2019_2666_MOESM1_ESM.docx]

**Additional file 1 Summary of data extraction by theme**

| **First author** | **Year** | **Understanding of malaria** | **Attitudes towards interventions** | **Risky behaviours** | **Treatment seeking** |
| --- | --- | --- | --- | --- | --- |
| Adhikari | 2018 | Respondents had a good understanding of malaria: able to clearly identify symptoms, understanding that illness could lead to death and aware that mosquitoes transmit the disease (in local language malaria = disease-mosquito bite/*aai moi kap* and ‘disease-fever with chills/*aai singyet*). One respondent linked cleanliness with low risk of infection. Respondents also had a certain understanding of asymptomatic malaria: only people in their community could have it and not outsiders. Respondents associated malaria with forest visits and rice fields, and poor clothing choices. | Although the respondents did not fully understand the reasoning behind MDA, they were supportive of the efforts and chose to participate to improve their health, which they viewed as poor. Respondents claimed that they would consent to taking medicines even if they did not present with symptoms for them to be healthy. After MDA, a participant reported telling other villagers about the project and that it was giving malaria medicine, which would be useful for poor people. | Respondents were aware of the increased risk of malaria during visits to the forest and rice fields. They were also aware of increased risk due to poor clothing choices and that wearing long-sleeved items of clothing would protect them. They also mentioned that sleeping under mosquito nets would offer protection. | Respondents said that if they suspect malaria they would go to the health centre to get a diagnosis and take the medicine prescribed to them by the doctor. However, they would not seek treatment at the health clinic if they did not have enough money, were unable to get to the centre either due to lack of transportation or difficulty in getting there due to the poor condition of the roads during the rainy season. After treatment, it was observed that patient demand for IV fluids increased as this was thought to restore their energy and blood levels. |
| Bannister-Tyrrell | 2018 |  |  | Bed net usage was high in forest farms to prevent insect bites but certain activities delayed sleeping times: drinking rice wine or fishing. Respondents also had different sleeping locations/patterns depending on their activities. If hunting at night, they would spend a single night at their plot hut, but if they were logging, they would spend many nights in the forest. |  |
| Chen | 2017 | Respondents reported that malaria was transmitted by mosquitoes, infected water or wind, and that only unhealthy people could get infected. Many described a low risk of malaria. There was limited understanding of the symptoms (cold-like) and treatment. Many had never taken the full course of drugs, stopping when symptoms were alleviated. Respondents said that they received only very little information on diagnosis and treatments. | Reported that they had very limited access to healthcare information. They normally got this information by word of mouth, community leaders and mobile phones but this was limited when they were in the forest. | Respondents reported using hammocks and bed nets to prevent mosquito bites but not consistently. Some described drinking alcohol to prevent bites. Bed nets were not used after people drank alcohol. Hammocks were not very common: unheard of or difficult to find (only available in Cambodia). People spend either a few days in the forest during the dry season but would spend up to a month at a time there sometimes. | Community health services were avoided due to extra costs for transportation and consultation, with treatment free to only those who have insurance. Pharmacies and private clinics were preferred over public facilities because of longer opening hours, friendlier staff and more convenient. Self-treatment was common, with anti-malarials obtained from friends, mobile vendors, pharmacies and grocery shops. They take the drugs, which were sometimes cocktails with them to the forest to treat their symptoms. Many buy the drugs in Cambodia where they are easier to obtain. |
| Crawshaw | 2017 | Respondents showed a good understanding of malaria: able to describe the symptoms and aware that mosquitoes transmit malaria. However, some still thought that malaria could be caused by poor hygiene practices, drinking/using dirty water and eating specific foods. | Most participants wore the distributed clothing regularly, particularly when working at night (a few participants wore the clothes during the day. They found that the clothing effectively prevented mosquito bites and appreciated the health benefits. Some participants reported that the clothing would now allow them to work longer hours without having to worry about insect bites. Respondents could not tell the difference between the insecticide treated and non-treated clothing, but had concerns about the potential health risk of the insecticide. Participants did not wear the clothes when washing/drying, if it was too hot or if it did not fit well. Participants stopped other preventative measures (wearing mosquito coils or repellents) while working because they found the clothing more effective. Due to the overall positive impression of the clothing, respondents recommended them to others in the community. Suggestions for further interventions recommended by the participants included advertising the clothing through promotional messages and the media, along with increasing the accessibility of the clothing by making them available in health facilities and shops (members of the community showed a willingness to buy the clothing). | There were some reservations about wearing long clothing to protect from mosquito bites. Younger generations preferred trousers, whereas older people preferred wearing *longyi/htamein* (female sarongs) during rubber tapping. However, respondents were less likely to wear long clothing during warmer weather, instead opting for shorts and short sleeves. Other precautions included clearing the area of stagnant water and cleaning water supplies (likely to prevent mosquitoes from breeding) and wearing mosquito coils in clothing (unconventional method) when working at night. Due to the smell, perceived toxic nature and risk of adverse side effects of mosquito repellents, they were not commonly used. |  |
| Grietens | 2010 |  |  | Many respondents had two homes: one in the village and one in the forest that they would sleep in when harvesting and during the rainy season. Households have enough ITNs (distributed during the NMCP), and non-treated nets purchased from the market. So they have sufficient nets in multiple locations. When in the deep forest, participants described sleeping in improvised ways in un-cleared portions of the forest (using hammocks). |  |
| Grietens | 2012 | There were misconceptions amongst respondents regarding how malaria was acquired. Causes included eating unsafe food, anaemia and poor hygiene. The use of a bed net was correctly identified as preventative. Respondents were aware of the increased risk of mosquito bites during the evening and early morning, however they reported that during the night there were few to no mosquitoes. Participants also viewed mosquitoes in the village as more dangerous than those in the forest: in the village there is more light and stagnant water resulting in more mosquitoes. They also described transmission as only happening in the village because there are people with malaria in the village. Some reported that forest mosquitoes did not cause malaria. |  | Bed net usage was irregular amongst participants, and when used this was not by all household members. There were many reasons for this: it is too hot to sleep under bed nets and when it is too cold, poorer participants who could not afford blankets would use the bed net instead. When moving between the forest and village, respondents would not necessarily take the bed net with them because this was either too much effort or would leave other members of the household without a net. |  |
| Grietens | 2015 |  | Respondents described that the disadvantage of using the LLIH was that it prevented families from sleeping together. However, they would be used during the day and evening, particularly by children. Hammock use was higher among people who slept alone (away from the family). | Many respondents had two homes, one in the village and the second in the fields located next to the forest. They slept in the latter when harvesting and during the rainy season. When sleeping in the deep forest, respondents would sleep on the floor and use blankets instead of bed nets or hammocks to keep warm and protect against mosquito bites (blankest were said to provide enough protection against mosquitoes). |  |
| Gryseels | 2013 | Although respondents had a good understanding of the cause, symptoms and methods of treating malaria, some reported that the disease could have supernatural causes. In such cases they would seek out diviners to treat them. |  |  | If malaria was expected, patients would first seek out the VMW. If they did not have RDTs or treatment they would then go to either the private or public health clinics. VMWs were perceived as unreliable because they were not always available and ran out of treatment and RDTs quickly, particularly during the rainy season when malaria burden is highest. There was a general preference for the public sector over the private sector. In the private sector they found that they would be seen quicker and were treated better by the staff, were able to select/avoid certain drugs (such as Malarine administered by VMW) that have pronounced side effects. However, in private facilities anti-malarial artesunate or chloroquine monotherapy were often administered in cocktails also containing other drugs – participants would buy as many as they could. Could also buy artemether injections (preferred over Malarine since offers faster relief and no side effects) in the private sectors – could be administered there or take away which was quite common, many did not take the full course because it is 5-day injection treatment which they could not afford. It was important for respondents to feel better as quickly as possible to go back to farm work and therefore preferred artemether injections. Cocktails are also quite popular because they do not provoke side effects and are cheaper than artemether injections. Some would seek treatment from diviner if they did not feel better after medicine. |
| Gryseels | 2015a |  | Due to the strong smell of the repellent, some respondents thought that it could be poison. However, others thought that the smell was important for the repellent to work. There was concern regarding its safety, particularly for children. The repellent was thought to be effective in repelling mosquitoes and other insects, and was even used against hair lice, leeches and maggots. Its use was sporadic – some people forgot to use it and its use was much lower in women and children compared to men who like to use it in the forest while hunting and fishing. Insecticide use was popular during hot nights when bed nets were not used and when the bed nets were being washed or repaired. |  |  |
| Gryseels | 2015b |  | Respondents thought that insects could get inside the LLIN/ITN, despite the insecticide treatment, depending on the brand of ITN/LLIN and the mesh size of the net. This was more common amongst people who used intact nets compared to those had torn nets. | Many respondents had two homes, one in the village and one in the fields located in the forest. They would sleep in the latter harvesting and during rainy season. The forest farm plot house consisted of wooden or bamboo houses on stilts. The young members of the family often slept outside of the house (to gain more independence) in hammocks and were more likely to not use bed nets and sleep in the forest fields and deep forests. |  |
| Gryseels | 2015c |  | Although market nets are not treated with insecticides, they are often favoured by respondents over LLINs. This is because market nets are larger, allowing bigger families to sleep together, and have smaller mesh sizes compared to LLINs which are thought to still allow small insects to penetrate despite being treated with insecticide. There was a difference in preference for each type of net, depending on the location with LLINs preferred in farm or rice field. | Many respondents had two homes, one in the village and one in the fields located in the forest where they sleep when harvesting and during rainy season. Mosquitoes are perceived to be more of a nuisance in the farms and deep forest than in the villages. Although respondents sleep shortly after sunset and do not engage in many activities in the evening, the houses in the forest farms houses are often partially open or are bamboo-thatched plot huts therefore providing minimal protection from insects in evening hours. During resting hours (which coincide with prime vector activity), many people rest and fall asleep without a net. These individuals are more likely to be children than adults and this happens more in the forest farm than village. During the night, people move to sleep under bed nets. Due to the lack of sanitary toilet facilities, many people urinate and defecate in the forest close to their house, which exposes them to mosquito bites. This also happens in the evening, night and early mornings when vectors are present. |  |
| Lim | 2017 | Those who had experienced malaria understood the seriousness of the disease and would consult their village malaria workers (VMWs) for diagnosis and treatment. They associated malaria with incapacity to work, reduced income and that it could result in death. Some have supernatural beliefs. | Due to their superstitions some villagers do not want blood tests or vaccinations. Iit was very difficult to reach forest-goers and migrants because they are away for long periods and unable to attend the event. However, some attendees said that they would advise their partners who go to the forest to use appropriate precautions, such as repellents to prevent malaria. The respondents said that because of the drama project they would start using repellents during the day in addition to the bed nets. They would also wear long clothing and bring mosquito nets to the forest and some respondents were more willing to do the blood tests. |  | Some villagers had concerns regarding blood tests because they either worried that healthcare workers would sell the blood. Some reported using supernatural preventative measures and therefore do not want blood tests or to be vaccinated. |
| Liverani | 2017 | All forest-goers described how malaria was transmitted by mosquitoes. However, a few also reported that it could also be caused by drinking contaminated water. Most were able to describe the symptoms of the disease and likewise most suspected malaria after they presented with symptoms, which would make them seek out the services of CHWs. The majority were also aware of RDTs as a diagnostic tool. Although some would seek treatment from traditional healers they would later realize that the treatment was not effective and therefore sought out VWMs or health clinics |  | Most patients who visited CHWs were young men involved in forest work. | Most respondents self-medicated, using purchased drugs or using traditional therapies, such as coining or using herbal medicines. If ineffective, they would then seek out treatment from either the public or private sector or both. Their choice depended on the availability of the health provider, their financial abilities amongst other factors. There was a preference for the private sector amongst respondents, due to the quick treatment offered and longer hours when compared to public services, such as CHWs who were not always available, had poor road accessibility or were unwilling to visit patients who lived far away. Furthermore, they would not go to the CHW if they suspected something other than malaria because they could get a more general diagnosis from other providers. Some also were unaware of where the VMW is located because they cannot read the signs. Some respondents admitted using traditional healers but after that treatment failed they would seek the proper diagnosis and treatment provided by CHWs. Forest-goers who contracted malaria in the forest self-medicated first but if ineffective would go back to the village to seek treatment. |
| Lyttleton | 2016 | Forest-goers report that forest activities increase their chances of getting malaria, even stating that everyone who has gone to cut rosewood has got malaria at some point. One respondent however suggested that karma could lead to infection. | Very rarely do forest goers attend mass screenings for malaria because they are worried that being diagnosed with malaria would indicate that they partook in criminal activities in the forest, or that the tests would show that they have drugs such as amphetamines in their system. | Many villagers go to the forest to cut down rosewood due to its high value. Although they are aware of the risk of malaria, that they are not discouraged because it is easy to get treatment. When in the forest, they work at night due to the illegal nature of their work. Because it gets very hot, they do not use repellents or wear long clothing and to repel mosquitoes they burn leaves. The use of alcohol and drugs in the forest also make them more unlikely to use effective preventative measures. | After returning from the forest, the group keep in contact to see if anyone has a fever. If one of them gets malaria in the forest, they assume all of them have been too. However, the risk of malaria does not deter them from going again. One respondent was not cured because he did not adhere to the medication, and was aware that that was the reason why he got sick again. He said that once he felt better it is easy to stop taking pills. |
| Panvisavas | 2001 | Respondents identified potential malaria treatments (including bitter parts of a tree) rather than preventative methods. Many are unable to describe the symptoms of the disease. They had a poor understanding of the mosquito and limited knowledge on the role of the mosquito in transmission. There is some understanding of the parasites, however, this is limited to the respondents being able to name *vivax* and *falciparum* with no understanding of what the parasites do. “Poverty deprives them of malaria knowledge, and it is because of poverty they are exposed to malaria.” |  | Due to the poor understanding of malaria and its transmission, many were unaware of the appropriate preventative measures. Many men describe alcohol as malaria prevention. Due to poverty, they are unable to afford mosquito nets and therefore use blankets and sarongs to protect themselves from mosquito nets. They are also unable to sue fire or smoke to repel mosquitoes. | Although blood tests and malaria clinics are free, they must pay for anti-malarials and treatment for severe cases in hospitals are very expensive. Because only those with identification cards can access welfare, those who do not have identification cards and are very poor can not seek appropriate treatment. |
| Pell | 2017 | Respondents had a good understanding of malaria and its symptoms, being able to list many of them correctly. Therefore, when they get malaria they can identify the disease based on the symptoms. They understood that it is transmitted by mosquitoes (knew it was the females) and could name the species (*vivax* and *falciparum*). Some misconceptions about aetiologies. | Although there was a fear of getting malaria, not all respondents adhered to the different rounds of MDA this is namely due to the side effects of the anti-malarials. Some even had to get IVs to counteract the side effects (and considered that too high an expense). Some did not want to participate because they were afraid of getting other diseases due to the blood collection component of the study and previous reports of the spread of HIV/AIDS through injections in another village in Cambodia. Villagers who were in the forest during the MDA showed poor adherence. There were some reports of people asking whether they could take the drugs to the forest with them – and for them to be made aware of when the next project would happen so that they could be back for it. | There was a good understanding of the different preventative measures, such as using bed nets, wearing long clothing and using fires in the forest to repel mosquitoes. The risk of malaria among forest-goers was understood, and this was associated with men because they were more likely to go to the forest than women. Forest-goers used bed nets less often when in the forest. Respondents reported being afraid of getting malaria and therefore adhering to good preventative measures such as bed net usage. | Respondents relied on CHWs for diagnosis and treatment, whereas, in the past, they said that they would get drugs from different sources. There was a good understanding of the fact that treatment consisted of multiple doses. |
| Sahan | 2017 | Respondents sometimes described malaria as a symptom instead of a disease. They could describe some of the symptoms of malaria and sometimes called the disease ‘forest-sick’ since they associated their illness with the forest but did not necessarily know why the forest made them sick. | It was difficult to reach some people who were absent for long period to work in the forests, rubber plantations and fields. | Although respondents reported that mosquitoes could transmit the disease, not many used LLINs because it was too hot. | Villagers used traditional medicine and healing practices to treat themselves and although they also used Western medicine, health staff thought that the traditional healing practices would reduce their willingness to use Western medicine. |
| Shafique | 2016 | Although respondents described how mosquito bites caused malaria, many reported that it could also be caused by forest spirits, unclean water and unhygienic surroundings. Those who went to the forest were considered a high-risk group. | Community members reported that the intervention improved the understanding of malaria and offered ways to prevent malaria. They voiced their support of continuing the project to further improve the understanding of villagers. Although the intervention stopped, PD sessions are still regularly held. There were observations/reports of improved preventative practices in village and seeking diagnosis and treatment directly from CHWs rather than the private sector. Forest goers also reported now using LLIHNs and wearing long clothing to prevent mosquito bites. | Many respondents wore long sleeves and burned mosquito coils to prevent bites. Although usage of LLINs was high, many of them had holes or were not used correctly. | After presenting with symptoms, respondents would first go to private drug sellers to self-medicate and only when their symptoms did not improve would they seek treatment from the health centre. Many also used traditional healing medicines and practices such as coining. Preference for private over public sector varied. Although public sector was preferred for its low cost, it was often described as unreliable and too far to access. Respondents would also be frustrated if they had symptoms and the CHW RDT tests would come back negative and could not offer treatment for anything. |
| Singhanetra-Renard | 1986 |  |  | When in the forest, villagers perform different activities (swidden farming, logging, hunting for boar, gaur, frogs and deer, foraging for bamboo shoots and rare plants, collecting honey, plants and medicinal herbs or smuggling cattle or poaching game). The activities tend to be illegal and therefore they are likely to perform them at night. Time spent in the forest depends on the activities: from a couple of days to a few weeks. Some do this as their main job; others supplement their farming jobs with foraging in the forest to make extra money. When in the forest, they take plastic sheet and a blanket, and they sleep under these – they do not take bed nets. |  |
| Taffon | 2018 | There were mixed responses regarding the cause of malaria amongst villagers. Some stated that *Anopheles* caused it, others described that it could relapse because of the food they had eaten or that they had done heavy work. Some respondents understood that the 3-day course should be taken, and if they did not that they would get sick again. Forests and plantations were identified as high-risk locations. Stagnant water was thought to attract a lot of mosquitoes along with dirty houses and environments. | There was a lack of participation due to people going to the forest. | Respondents described the importance of wearing long clothing and sleeping under mosquito nets to prevent getting malaria and that it was not sufficient to prevent malaria by burning a fire. Although the risk of getting malaria in the forest was understood, some respondents ignore the risk and still go because the forest provides them with food. Men, women and children all go to the forest farms. |  |
| Verschuere | 2017 | ﻿ ﻿Although almost everyone knows that malaria is caused by mosquito bites some think that there can be additional underlying factors such as ghost attacks, sorcery, offending the deity of the forest, etc. They also think that malaria relapses can be caused by eating the wrong types of food and that when someone is sick with malaria they should avoid certain foods. Environmental locations are associated with malaria such as the forest and also areas with poor sanitation and stagnant water where mosquitoes could breed |  |  | Depending on the malaria diagnosis (*falciparum* or *vivax*) different health seeking patterns are observed. With *vivax* they buy medicines while *falciparum* or mixed would go to the hospital. People also seek out home treatments such as traditional medicine and practices such as fanning, coining, or fever showering. This depends on what the symptoms are. Some take biomedicines such as paracetamol to relieve fever symptoms. People can also buy drug cocktails from the private clinic which they take at home. Other people will look for VMW or doctor test and give treatment. Most people think that injections (IV or ampules) are more effective than taking tablets and patients are more likely to get this type of treatment in the private clinic. If they do not get better after seeking different treatments will try spiritual or sorcery-related treatments. The financial situation played an important role in the where the respondent went for treatment. Some respondents said they will try to self-treat with biomedicines then if that does not work go to the health centre since it costs money to get there. The health clinics weren’t very favourable due to the long waiting times and the fact that they were not always available. The private clinic is perceived as providing better services |
| Wharton-Smith | 2014 | Malaria is considered a common disease amongst forest-goers, and amongst community members going to the forest is considered one of the biggest risk factors for getting malaria. The understanding of malaria transmission varied. Although there was an understanding that malaria could be transmitted by mosquitoes, an equal number thought it could be caused by drinking or bathing in contaminated water. Therefore, some respondents would avoid drinking un-boiled water or taking baths, but highlighted this as an issue in forest areas. Other common beliefs were that malaria could be contracted when doing heavy labour or feeling tired. Participants were aware that there were more mosquitoes living in forested areas than in the villages. | Bed nets are too rough and difficult to pack, the holes are too big and let insects inside, they prefer smaller single size nets in the forest, the smell of the LLIN is strong or irritates their skin making them feel itchy. Instead wanted easily portable nets, with softer texture that would be easy to hang and did not have a smell but were long lasting. One respondent said that the distribution should be increased specially since the nets are expensive and they cannot afford them. Respondents also complained that they were not receiving adequate health information on malaria. Some said they had never received any information since no one comes to visit them on the forest or because they do not have access to TV and radio. They would like to get more information. | Using smoke to deter mosquitoes was the most common measure followed by wearing long clothing, however, respondents would take off long clothing after working if they got too hot or some thought it did not offer complete protection since they could still get bitten when wearing long clothing. Mosquito coils were also commonly used to deter mosquitoes, but respondents were concerned about their health due to the smoke. Blankets were used instead of bed nets by some respondents to protect from bites. Repellents were said to be used, but this was not a common method and was not adhered to. Reasons for not using repellents included the fact that they were not viewed as being effective, were toxic and were too expensive. Those who did use repellents thought they were a good way of protecting themselves when bed nets could not be used. Hand fans were also used to prevent mosquito bites. There was a misconception that drinking alcohol has a protective effect, preventing mosquitoes from biting. Hammock nets were not a common prevention method, but the participant who did use it said that hammocks are much more convenient in the forest than bed nets, but only those who could afford them would use them (implying that they are expensive). Mixed responses/behaviours in terms of bed net usage. Respondents did not think nets were sufficient to prevent mosquito bites, particularly when working during the day but thought they were good at preventing bites at night or even for evening activities. Not all respondents had bed nets or LLINs, as they were not present when they were distributed. For some forest goers said even though they owned bed nets they would not bring them to the forest because they were inconvenient to bring (too bulky) or difficult to hang or had no space for them. Some used the bed nets for other purposes like as a pillow or a blanket during cold nights. One respondent even said that he would only bring the bed net if staying for longer than one or two nights in the forest. Some did not want to bring the net since that would leave their family without a net in the village. Some did not sleep under the net because it irritated their skin or made them feel too hot. Forest goers were more likely to use non-impregnated nets purchased from the market rather than LLINs either because LLINs were too expensive and market nets were cheaper or because they preferred market nets because of the net properties. Awareness that not sleeping under bed nets in the forest leads to mosquito bites and getting malaria, but respondents still chose to sleep without a bed net, but some were not aware of how to prevent so did not use a net. Some people did not use any preventative measures, either because they did not know what to do or because they though it is easy to take medicines once they contract malaria so did not see it as a risk. Things done in the forest: Searching for gold, gather food, cut bamboo or firewood. |  |
